# Successful Treatment of an Infant with Left Ventricular Noncompaction Presenting with Fatal Ventricular Arrhythmia Treated with Cardiac Resynchronization Therapy and an Implantable Cardioverter Defibrillator

**DOI:** 10.1155/2018/9562326

**Published:** 2018-04-01

**Authors:** Masato Kimura, Kengo Kawano, Hisao Yaoita, Shigeo Kure

**Affiliations:** Department of Pediatrics, Tohoku University Graduate School of Medicine, 1-1 Seiryo-machi, Aoba-ku, Sendai, Miyagi 980-8574, Japan

## Abstract

We herein report the successful treatment of a 4-year-old girl with left ventricular noncompaction (LVNC) who presented with incessant ventricular fibrillation at 5 months of age. An implantable cardioverter defibrillator (ICD) was implanted, and dual chamber (DDD) pacing was initiated at 7 months of age. At her 10-month follow-up, her left ventricular ejection fraction (LVEF) had decreased from 45% to 20% with mechanical dyssynchrony. After upgrading to cardiac resynchronization therapy (CRT), the LVEF improved to 50%. The usefulness of CRT in pediatric LVNC has not been fully elucidated. However, our case suggests that CRT therapy may be an effective option for LVNC-induced cardiac dysfunction.

## 1. Introduction

Left ventricular noncompaction (LVNC) is a rare congenital cardiomyopathy with a clinical presentation that ranges from no symptoms to sudden cardiac death due to fatal arrhythmia or heart failure. The onset of fatal ventricular arrhythmia under 1 year of age is a risk factor for cardiac death and an indication for heart transplantation [1]. LVNC is characterized by prominent left ventricular (LV) trabeculations and deep intertrabecular recesses, which are assumed to be the result of failure or abnormality of the myocardial compaction process during the prenatal period [2]. However, Jensen et al. recently hypothesized that hypertrabeculation may not be a failure of myocardial compaction, but rather an aberration of mammalian cardiogenesis [3]. Cardiac resynchronization therapy (CRT) for heart failure in pediatric congenital heart disease is an emerging option [4, 5]; however, few reports on CRT in pediatric LVNC patients are available [6].

## 2. Case Report

**Figure 1:**
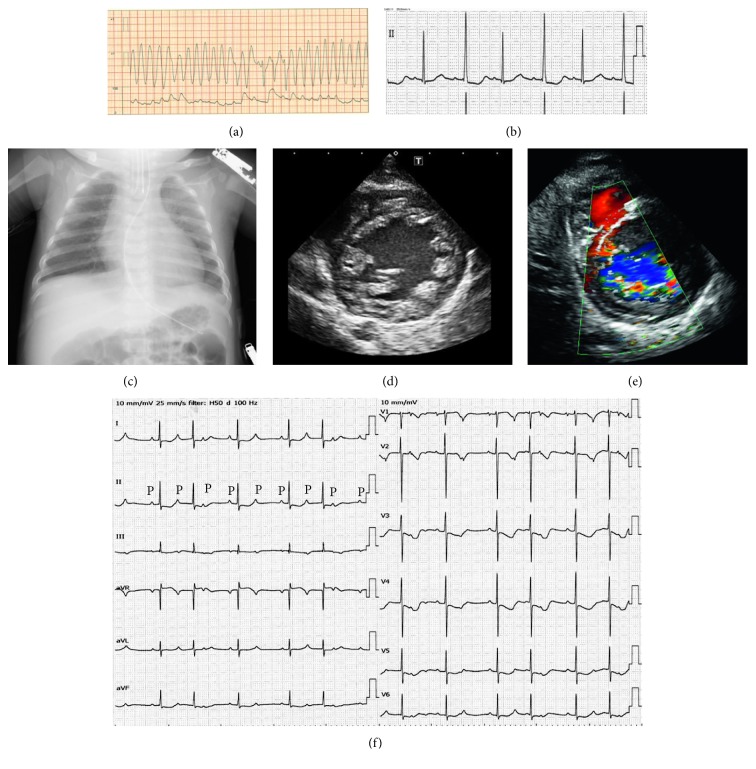
Electrocardiogram (ECG) and arterial blood pressure monitoring on admission (a). (Top panel) ECG shows polymorphic ventricular tachycardia, with a rate of approximately 300 bpm. (Bottom panel) arterial blood pressure monitoring. (b) Lead II of the ECG after polymorphic ventricular tachycardia was stopped. The heart rate (HR) was 79 bpm, PR interval was 160 msec, and QTc interval was 510 msec with alternate T waves. (c) Chest X-ray on admission showing a cardiothoracic ratio of 52%. Transthoracic echocardiography on admission. (d) Transthoracic echocardiography shows left ventricular noncompaction on the parasternal short axis view, with a ratio of noncompacted to compacted area at end-systole > 2. (e) Color Doppler imaging reveals communication between myocardial recesses and the left ventricular cavity. (f) The 12-lead ECG before implantable cardioverter defibrillator (ICD) implantation, showing 2 : 1 atrioventricular block. The HR was 76 bpm, PR interval was 170 msec, and QTc interval was 462 msec. P indicates the P wave in lead II.

A 6-month-old girl was transferred to our university hospital presenting with incessant ventricular fibrillation (VF) after rewarming therapy for cerebral hypothermia. There was no family history of cardiovascular or neurologic disease. Her development until the event was normal, and at 5 months after birth, she went into cardiopulmonary arrest. During cardiopulmonary resuscitation by emergency medical services, VF was detected using an automated external defibrillator. Sinus rhythm was restored by defibrillation shock. Cerebral hypothermia treatment for 4 days was initiated at a tertiary emergency city hospital. After rewarming, VF occurred again and incessantly repeated without stimulation. She was moved to our university hospital, and her electrocardiogram (ECG) showed polymorphic ventricular tachycardia and VF (Figure 1(a)). After administration of various antiarrhythmic drugs and sedatives, the fatal arrhythmia finally stopped (Figure 1(b)). Laboratory blood tests did not indicate myocarditis or metabolic disease. A chest X-ray revealed no cardiomegaly with a cardiothoracic ratio (CTR) of 52% (Figure 1(c)). Transthoracic echocardiography confirmed global LV hypokinesis with an ejection fraction (EF) of 30%. B-mode analysis revealed a two-layer structure with a compacted thin epicardial band and a thick, noncompacted endocardial layer with prominent trabeculae and deep intertrabecular recesses at the LV apical and lateral walls. In the end-systolic phase, the ratio of noncompacted to compacted layers was 2.8. Color Doppler imaging revealed blood filling the recesses from the ventricular cavity (Figures 1(d) and 1).

**Figure 2:**
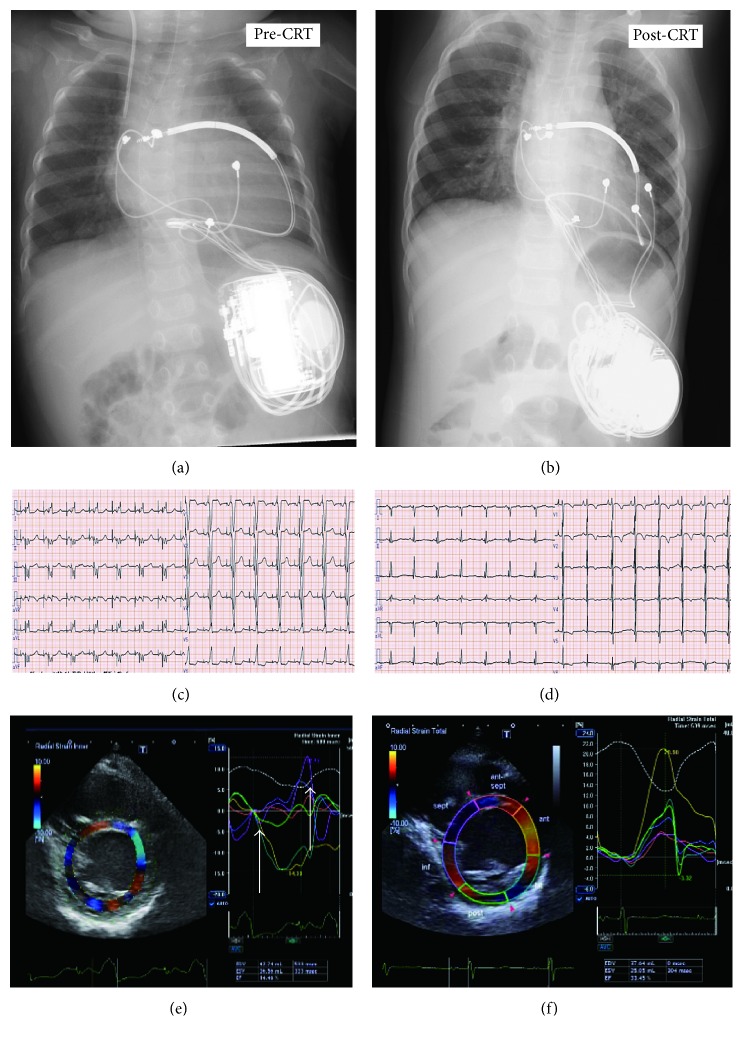
Chest X-rays pre- and postcardiac resynchronization therapy (CRT). (a) The defibrillator was placed into the left upper abdomen, and the shock lead was fixed at the level of the superior vena cava. The right ventricular (RV) epicardial lead was placed at the RV free wall. (b) The left ventricular (LV) lead was placed at the LV lateral wall. The 12-lead ECG pre- and post-CRT. Before CRT (c), the QRS duration was 114 msec. After CRT (d), the QRS duration was shortened to 80 msec. Speckle-tracking radial dyssynchrony pre- and post-CRT (Artida™; Toshiba Medical Systems, Tokyo, Japan). Pre-CRT (e), dyssynchrony is shown as a time difference (arrow) between time to peak strain in the anterior wall (yellow) and septum peak strain (purple) (dual chamber (DDD) 90–150 bpm with an atrioventricular (AV) delay of 130 msec). Post-CRT (C-II), dyssynchrony disappeared (DDD 80–160 bpm, with an AV delay of 120 msec and a LV-RV delay of 0 msec).

For secondary prevention, an implantable cardioverter defibrillator (ICD) (Protecta™ XT DR; Medtronic, Minneapolis, MN, USA) was implanted in the patient's left upper abdominal quadrant, and dual chamber (DDD) pacing was started due to 2 : 1 atrioventricular (AV) block (Figure 1(f)), which might have been induced by administration of amiodarone and beta-blocker (at 7 months of age; height 68 cm, weight 5.6 kg) (Figure 2(a)). The shock lead was run outside of the epicardium and fixed at the level of the superior vena cava. At discharge, the left ventricular ejection fraction (LVEF) was 45% and the brain natriuretic peptide (BNP) level was 96 pg/ml. At her 10-month follow-up, the LVEF decreased to 20% and the patient's mitral regurgitation had deteriorated severely. Moreover, mechanical dyssynchrony was observed on strain echocardiography (Figure 2(e)), and the patient's BNP levels increased to 1,300 pg/ml. After ICD implantation, appropriate discharge of the ICD occurred only once when she had developed acute gastroenteritis with ventricular tachycardia. ICD right ventricular (RV) pacing was upgraded to biventricular pacing with a cardiac resynchronization therapy defibrillator (CRTD) (Viva™ XT; Medtronic, NJ, USA) (height 72 cm, weight 8.5 kg). After this upgrade, her LV function gradually improved, with an LVEF of 50% without mechanical dyssynchrony and mild mitral regurgitation (Figure 2(f)). The BNP level decreased to 26 pg/ml, and the CTR was 53% (Figure 2(b)). Over a 3-year follow-up period, the patient's course was uneventful and she was maintained on bisoprolol (0.3 mg/kg), enalapril (0.25 mg/kg), aspirin, and diuretics. The extent and location of abnormal LV trabeculation did not change during this period. Her developmental delays improved, with exercise, social, and vocal capacities being at the 2-, 3-, and 3-year-old levels, respectively.

Whole exome sequencing revealed a nonsense mutation in exon 7 of the *DTNA* gene (C601T). While the patient's mother also had the same mutation in the *DTNA* gene, echocardiogram revealed that the mother's heart was morphologically and functionally normal. The patient's father did not have a mutation of the *DTNA* gene. The *DTNA* gene of the patient's older sister was not investigated because of her normal echocardiogram with no medical history.

## 3. Discussion

We herein report the successful treatment of a 4-year-old girl who presented with incessant VF and LVNC at 5 months of age. After RV pacing, her cardiac function deteriorated and LVEF decreased; however, CRT corrected her mechanical dyssynchrony, which subsequently improved cardiac function.

LVNC was first described pathologically by Grant in 1926 [7] and has been defined as a genetic cardiomyopathy by the American Heart Association since 2006 [8]. It is a rare disorder that is believed to develop due to cessation of the refinement of ventricular muscles between the 5th and 8th week of gestational age [2]. However, Jansen et al. recently hypothesized that hypertrabeculation is not a failure of myocardial compaction, but rather an aberration of mammalian cardiogenesis [3]. They proposed that the evolutionary and developmental models of normal and hypertrabeculated hearts and trabeculations in LVNC are not similar to the trabeculations of embryos or ectotherms, viewed in the light of histological and magnetic resonance imaging (MRI) findings.

The clinical presentation of LVNC ranges from no symptoms to sudden cardiac death due to fatal arrhythmia or heart failure. Brescia et al. investigated the long-term prognosis of 242 pediatric LVNC patients and reported that the mortality rate was 12.8%, the sudden cardiac death rate was 6.2%, and 5.4% of patients received a heart transplant [1]. The risk of death was strongly associated with cardiac dysfunction and ventricular arrhythmias for <1 year. LVNC is a genetically heterogeneous disorder, and gene mutations, which encode sarcomeric, cytoskeletal, and nuclear membrane proteins, are identified in only half of all reported cases [2]. In 2001, Ichida et al. reported a family with LVNC and a *DTNA* missense mutation in exon 4 (C362T) [9]. Whole exome sequencing in our patient revealed a nonsense mutation in exon 7 of the *DTNA* gene (C601T). Interestingly, our patient's mother also had the same mutation; however, her heart was morphologically normal. These differences may be due to incomplete penetrance of the *DTNA* gene in our case and/or the dominant negative effects of the missense mutation in exon 4. To the best of our knowledge, a *DTNA* gene mutation associated with LVNC was observed in only one family, and the function of the *DTNA* gene in LVNC has not been fully elucidated. No specific treatment has been established for LVNC, and ICD implantation is performed as a preventive measure for lethal ventricular arrhythmia. In a previous study, Kobza et al. reported that ICD implantation was effective as a primary and secondary preventive measure for sudden cardiac death in LVNC patients (30 patients; mean age 48 ± 14 years) [10]. Moreover, ICD with cardiac resynchronization improved LVEF from 22 ± 5% before implantation to 37 ± 15% after implantation, with a mean follow-up of 18 ± 11 months (six patients; mean age 58 ± 17 years). These studies were conducted in adult populations. However, implantation of ICD in pediatric patients is associated with several limitations. Indeed, the size of the devices and lead implantation result in placement difficulties of these therapeutic devices in pediatric patients. In our case, it seems that RV (DDD) pacing using an ICD caused LV function deterioration due to mechanical dyssynchrony. Cardiac function dramatically improved after introducing biventricular pacing using CRTD. It is known that in a certain proportion of pediatric patients with RV pacing, LV failure may develop. Indeed, Roman et al. demonstrated that epicardial RV free wall pacing is a risk factor for pacing-induced heart failure [11]. Moreover, due to their small heart size, selecting pacing lead positions is very difficult in infants. RV pacing-induced LV dysfunction responds well to biventricular pacing therapy [4]. Currently, there are no prospective randomized controlled trials evaluating pediatric CRT, and device therapy has become an emerging option for treating heart failure in pediatric heart disease [5]. Most reports in the medical literature concerning the use of CRT in pediatric patients are case reports that focus on the efficacy of CRT in treating severe heart failure. Only two large studies on the use of CRT in pediatric patients with dilated cardiomyopathy (DCM) have been published. Dubin et al. [12] reported their findings in 16 patients, while Cecchin et al. [13] included 10 patients with DCM in their study. No mortality was reported in these studies; however, additional reports of similar cases are needed to determine standard guidelines concerning the use of CRT in pediatric patients. The usefulness of CRT in pediatric LVNC remains to be fully elucidated. Our case suggests that CRT therapy could be an effective option for LVNC-induced cardiac dysfunction.

The present case provides important insights into the midterm effects of biventricular pacing and CRTD implantation for infantile LVNC. It is necessary to consider pediatric body size with respect to device and lead implantation. CRTD treatment is useful for the management of infantile LVNC with mechanical dyssynchrony and may improve prognosis.
